# Pneumopéritoine post-traumatique révélant une perforation d’un diverticule de Meckel

**DOI:** 10.11604/pamj.2016.25.12.9414

**Published:** 2016-09-19

**Authors:** Messaoudi Ikram, Hedfi Mohamed

**Affiliations:** 1 Service de Chirurgie Générale, Hôpital des FSI, La Marsa, Tunisie

**Keywords:** Diverticule, Meckel, perforation, péritonite, Diverticulum, Meckel, perforation, peritonitis

## Image en médecine

Le diverticule de Meckel est une affection rare qui touche 2% de la population générale. Le plus souvent asymptomatique, le diverticule de Meckel peut se manifester par des douleurs de la fosse iliaque droite mais il peut aussi se révéler parfois chez l'enfant ou l'adulte jeune par des complications : occlusion, infection dite diverticulite, hémorragie digestive basse.la perforation est le plus souvent la conséquence d'un corps étranger intradiverticulaire. Nous rapportons le cas d'un patient âgé de 43 ans admis en urgence pour un syndrome péritonéal dans les suites d'une contusion abdominal (piéton heurté par une voiture), le scanner abdominal avait objectivé un épanchement de moyenne abondance avec un pneumopéritoine (A). Au terme de cette étape clinique et radiologique l'existence d'une perforation d'un organe creux en particulier colique ou duodénale a été suspectée, l'hypothèse d'une perforation grélique était faible car cela ne s'associe pas à un pneumopéritoine après quelques heures dans les suites d'un traumatisme fermé de l'abdomen. L'exploration chirurgicale avait montré une péritonite stercorale par perforation d'un diverticule de Meckel (B) sans autres lésions associés; il a été réalisé une toilette péritonéale avec une résection du grêle emportant le diverticule, une double stomie a la Bouilly volkman au niveau de la fosse iliaque droite. Les suites immédiates étaient favorables, le patient a été repris 3 semaines plus tard pour rétablissement de la continuité digestive. L'examen anatomopathologique de la pièce avait confirmé l'existence d'un diverticule de Meckel perforé.

**Figure 1:**
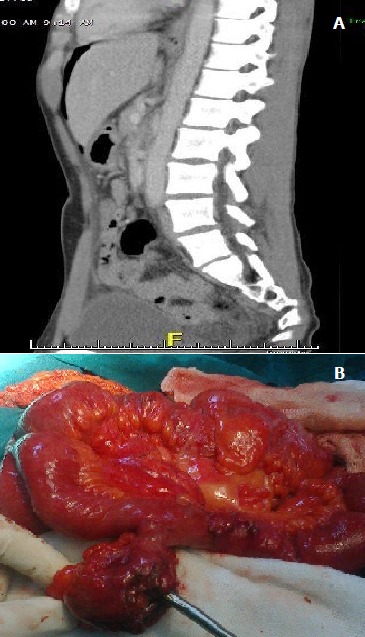
A) TDM abdominale en coupe sagittale pneumopéritoine avec épanchement liquidien dans le douglas; B) en peropératoire: perforation sur le sommet du diverticule

